# Clinical Significance of Preoperative Neutrophil Lymphocyte Ratio versus Platelet Lymphocyte Ratio in Patients with Small Cell Carcinoma of the Esophagus

**DOI:** 10.1155/2013/504365

**Published:** 2013-09-05

**Authors:** Ji-Feng Feng, Ying Huang, Qiang Zhao, Qi-Xun Chen

**Affiliations:** ^1^Department of Thoracic Surgery, Zhejiang Cancer Hospital, No. 38 Guangji Road, Banshan Bridge, Hangzhou 310022, China; ^2^Department of Operating Theatre, Zhejiang Cancer Hospital, No. 38 Guangji Road, Banshan Bridge, Hangzhou 310022, China; ^3^Key Laboratory Diagnosis and Treatment Technology on Thoracic Oncology, Zhejiang Province, Hangzhou 310022, China

## Abstract

Recent studies have shown that the presence of systemic inflammation correlates with poor survival in various of cancers. The aim of this study was to determine the prognostic values of neutrophil lymphocyte ratio (NLR) and platelet lymphocyte ratio (PLR) in patients with small cell carcinoma of the esophagus (SCCE). Preoperative NLR and PLR were evaluated in 43 patients with SCCE from January 2001 to December 2010. The prognostic significance of both markers was then determined by both uni- and multivariate analytical methods. Receiver operating characteristic (ROC) curves were also plotted to verify the accuracy of NLR and PLR for survival prediction. Patients with PLR ≥150 had significantly poorer (relapse-free survival) RFS and (overall survival) OS compared to patients with PLR <150. However, RFS or OS did not differ according to NLR categories (<3.5 and ≥3.5). The areas under the curve (AUC) indicated that PLR was superior to NLR as a predictive factor. The results of the present study conclude that PLR is superior to NLR as a predictive factor in patients with SCCE.

## 1. Introduction

Esophageal cancer (EC) is the eighth most common cancer worldwide [1]. In China, the crude mortality rate of EC was 15.2/100,000, which represented 11.2% of all cancer deaths and ranked as the fourth most common cause of cancer death [2]. The most common histological types are squamous cell carcinoma and adenocarcinoma. Other histological types are uncommon, with small cell carcinoma being especially rare.

Small cell carcinoma of the esophagus (SCCE) is a rare disease, which was first described in 1952 by McKeown [3]. The incidence of SCCE between all esophageal malignancies is from 0.05 to 2.4% in western populations, and this rate rises up to 7.6% in Chinese and Japanese literature [4, 5]. Although advances have occurred in the multidisciplinary treatment in SCCE, the survival is still poor [5]. Therefore, assessing the prognostic factors in SCCE patients will become more and more important.

Recently, there is increasing evidence that a systemic inflammatory response is of prognostic value in various of cancers [6, 7]. C-reactive protein is an index of systemic inflammation. However, C-reactive protein is not routinely measured as part of preoperative examination. The neutrophil to lymphocyte ratio (NLR) and platelet to lymphocyte ratio (PLR) are other markers, and some studies have shown NLR or PLR to be a significant prognostic factor in cancers, including EC [8–10]. However, no studies regarding the predictive value of NLR or PLR in SCCE are available. Therefore, the aim of this study was to determine the prognostic value of NLR and PLR in patients with SCCE.

## 2. Patients and Methods

### 2.1. Patients

From January 2001 to December 2010, a retrospective analysis was conducted of 43 patients with SCCE who underwent curative esophagectomy at Zhejiang Cancer Hospital (Hangzhou, China). All of the patients included in the analysis fit the criteria: (1) SCCE confirmed by histopathology; (2) limited disease without distal metastasis; (3) curative esophagectomy with margins free of disease. All of the above patients were followed up by posting letters or by telephone interviews. The last followup was on November 30, 2011. All subjects gave written informed consent to the study protocol, which was approved by the Ethical Committees of Zhejiang Cancer Hospital, Hangzhou, China.

### 2.2. NLR and PLR Evaluation

Data on preoperative blood cell counts were extracted in a retrospective fashion from the medical records. All white blood cell and differential counts were taken within 1 week prior to surgery. The NLR was defined as the absolute neutrophil count divided by the absolute lymphocyte count, and it was categorized into two groups [11] (<3.5 and ≥3.5); similarly, PLR was defined as the absolute platelet count divided by the absolute lymphocyte count, and it was also categorized into two groups [10] (<150 and ≥150).

### 2.3. Statistical Analysis

Statistical analysis was conducted with SPSS 17.0 (SPSS Inc., Chicago, IL, USA). The Pearson Chi-squared test was used to determine the significance of differences for patients grouped by NLR and PLR. The relapse-free survival (RFS) and overall survival (OS) were calculated by the Kaplan-Meier method, and the difference was assessed by the log-rank test. Multivariate analyses were performed to evaluate the prognostic parameters for RFS and OS. Receiver operating characteristic (ROC) curves were also plotted to verify the accuracy of NLR and PLR for RFS and OS prediction. A *P* value less than 0.05 was considered to be statistically significant.

## 3. Results

**Figure 1:**
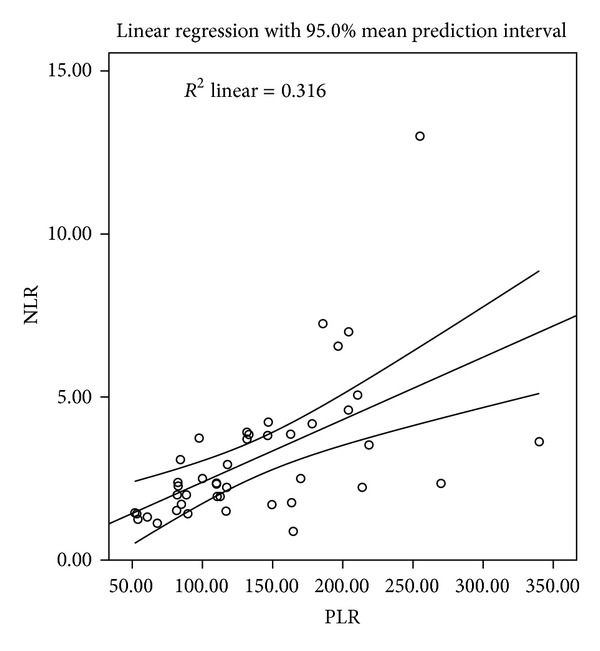
Correlation between the NLR and PLR (*r* = 0.563, *P* < 0.001).

Among the 43 patients, 13 (30.2%) were women and 30 (69.8%) were men. The mean age was 58.7 ± 7.8 years, with an age range from 45 to 74 years. All patients were treated with radical resection. Adjuvant chemoradiotherapy was used in 26 cases where 13 cases with four to six courses of platinum-based combination chemotherapy, 8 cases with radiotherapy, and 5 cases with chemoradiotherapy, respectively. All the clinicopathologic characteristics were comparable between patients grouped by NLR or PLR, as shown in Table 1. In addition, there was a positive correlation between the NLR and PLR (*r* = 0.563, *P* < 0.001) (Figure 1).

**Figure 2:**
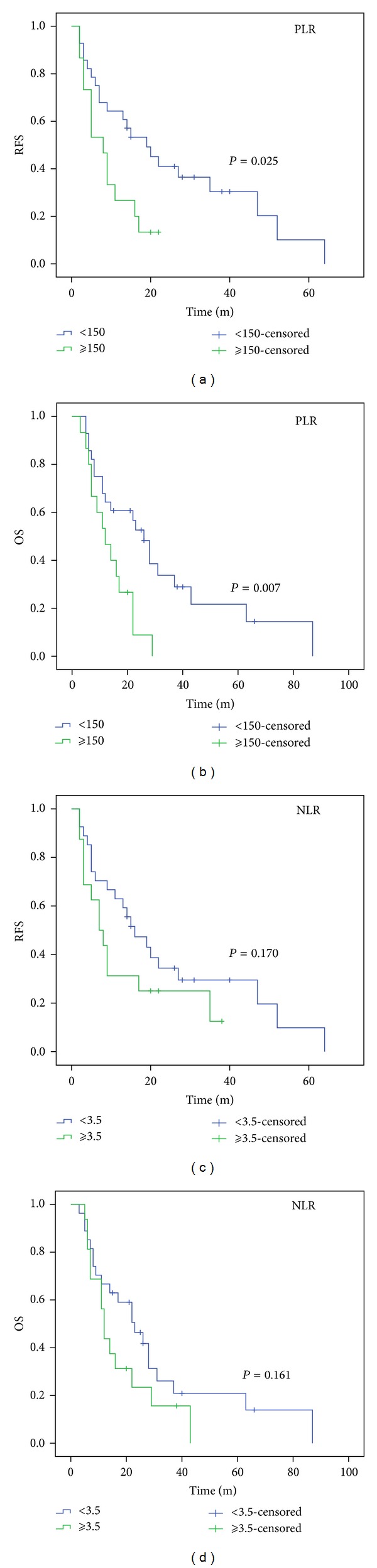
RFS (a) and OS (b) in PLR categories. RFS (c) and OS (d) in NLR categories. Patients with PLR ≥150 had significantly poorer RFS (13.3% versus 25.0%, *P* = 0.025) (a) and OS (6.7% versus 25.0%, *P* = 0.007) (b) compared to patients with PLR <150. However, RFS (22.2% versus 18.8%, *P* = 0.170) (c) and OS (22.2% versus 12.5%, *P* = 0.161) did not differ according to NLR categories ((c) and (d)).

Patients with PLR ≥150 had significantly poorer RFS (13.3% versus 25.0%, *P* = 0.025) and OS (6.7% versus 25.0%, *P* = 0.007) compared to patients with PLR <150 (Figures 2(a) and 2(b)). However, RFS (*P* = 0.170) and OS (*P* = 0.161) did not differ according to NLR categories (Figures 2(c) and 2(d)). PLR was a significant predictor of OS (*P* = 0.041) but not of RFS (*P* = 0.083) (Tables 2 and 3).

**Figure 3:**
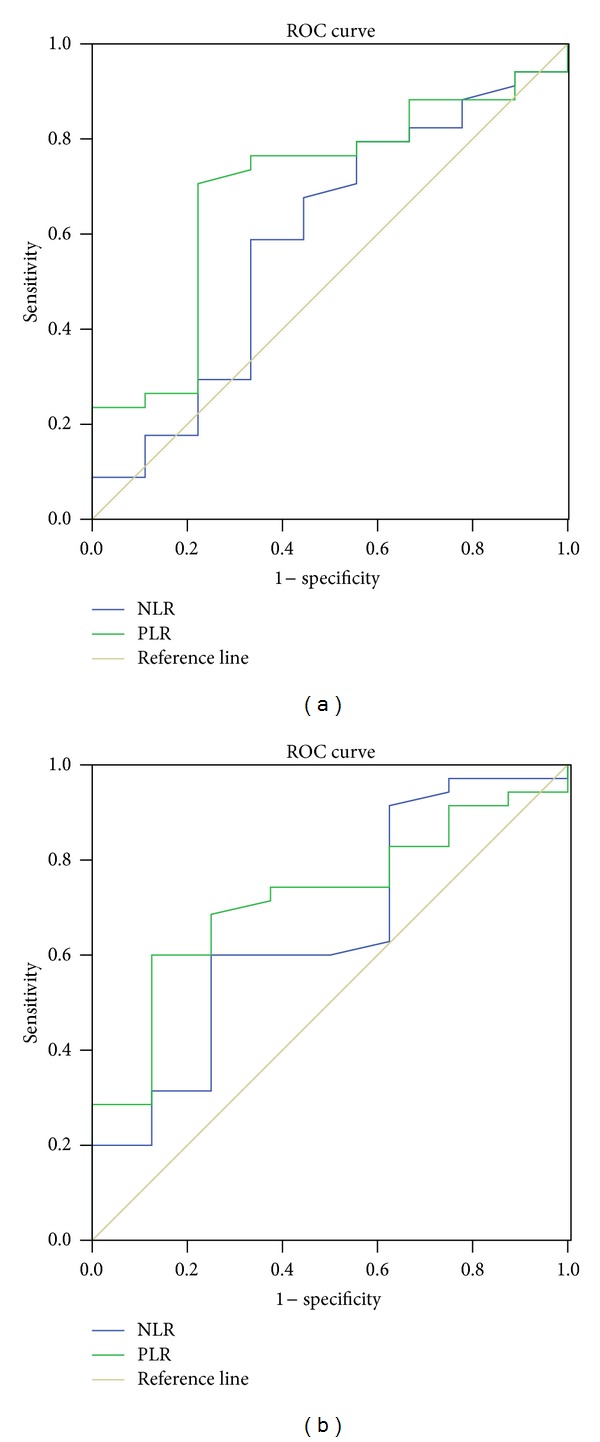
ROC curves for RFS (a) and OS (b) prediction. ROC curves were plotted to verify the accuracy of NLR and PLR for RFS and OS prediction. The AUC were 0.588 for NLR and 0.694 for PLR according to RFS prediction (a). The AUC were 0.650 for NLR and 0.720 for PLR according to OS prediction (b).

ROC curves were plotted to verify the accuracy of NLR and PLR for RFS and OS prediction. The areas under the curve (AUC) were 0.588 (RFS) and 0.650 (OS) for NLR and 0.694 (RFS) and 0.720 (OS) for PLR, indicating that PLR was superior to NLR as a predictive factor in patients with SCCE (Figure 3).

## 4. Discussion

To our knowledge, this is the first study to determine the prognostic value of preoperative NLR and PLR in predicting prognosis for patients with SCCE. Our study showed that there was a positive correlation between the NLR and PLR (*r* = 0.563, *P* < 0.001). Patients with PLR ≥150 had significantly poorer RFS and OS compared to patients with PLR <150. PLR was a significant predictor of OS (*P* = 0.041) but not of RFS (*P* = 0.083). The AUC were 0.588 (RFS) and 0.650 (OS) for NLR and 0.694 (RFS) and 0.720 (OS) for PLR, indicating that PLR was superior to NLR as a predictive factor in patients with SCCE.

There is strong linkage between inflammation and cancer [6, 7]. Cancer-related inflammation causes suppression of antitumor immunity by recruiting regulatory T cells and activating chemokines, which results in tumor growth and metastasis. The presence of both neutrophilia and thrombocytosis tends to represent a nonspecific response to cancer-related inflammation [12]. However, the mechanism between preoperative leukocytosis and neutrophilia and cancer remains unclear. However, cancer has been shown to produce myeloid growth factors, such as granulocyte colony-stimulating factor, tumor necrosis factor-alpha, interleukin-1, and interleukin-6, which may influence tumor-related leukocytosis and neutrophilia [13, 14].

Preoperative NLR is inversely related to prognosis in many cancers however, its role in EC is still controversial. Sato et al. [8] and Sharaiha et al. [9] demonstrated that a high NLR is associated with tumor progression and poor survival in patients with EC. However, Dutta et al. [15] and Rashid et al. [11] showed that NLR does not correlate with prognostic factor in EC. Moreover, there have been few studies available regarding PLR in EC patients. Dutta et al. [15] showed that PLR does not correlate with prognostic factor in patients with EC. In our study, however, patients with PLR ≥150 had significantly poorer RFS and OS compared to patients with PLR <150. By multivariate analyses, PLR was a significant predictor of OS (*P* = 0.041) but not of RFS (*P* = 0.083).

In the present study, the correlation between NLR and PLR was determined. As expected, we found that there was a positive correlation between the NLR and PLR (*r* = 0.563, *P* < 0.001). Finally, ROC curves were also plotted to verify the accuracy of NLR and PLR for survival prediction. The AUC were 0.588 (RFS) and 0.650 (OS) for NLR and 0.694 (RFS) and 0.720 (OS) for PLR, indicating that PLR was superior to NLR as a predictive factor.

There potential limitations of the present study include the relatively small number of patients as well as the fact that the analysis was retrospective and the mean follow-up duration was short. Furthermore, due to the limited number of patients with SCCE, our analysis may suffer from type I or type II error. The results of the study should therefore be regarded with caution. Larger prospective studies will need to be performed to confirm these preliminary results.

In conclusion, preoperative PLR was a significant predictors of OS in patients with SCCE. PLR is superior to NLR as a predictive factor in patients with SCCE.

## Figures and Tables

**Table 1:** The characteristics of the 43 SCCE patients grouped by NLR and PLR.

	Cases (*n*, %)	NLR (*n*)		*P* value	PLR (*n*)		*P* value
		<3.5	≥3.5		<150	≥150	
Gender				0.108			0.471
Female	13 (30.2)	11	2		10	3	
Male	30 (69.8)	16	14		18	12	
Age (years)				0.013			0.126
≤60	24 (55.8)	19	5		18	6	
>60	19 (44.2)	8	11		10	9	
Tumor length				0.018			0.407
≤3	18 (41.9)	15	3		13	5	
>3	25 (58.1)	12	13		15	10	
Tumor location				0.847			0.856
Upper/middle	25 (58.1)	16	9		16	9	
Lower	18 (41.9)	11	7		12	6	
Vessel involvement				0.835			0.993
Negative	33 (76.7)	21	12		22	11	
Positive	10 (23.3)	6	4		6	4	
Depth of invasion				0.278			0.139
T1-2	18 (41.9)	13	5		14	4	
T3-4a	25 (58.1)	14	11		14	11	
Nodal metastasis				0.018			0.408
Negative	15 (34.9)	13	2		11	4	
Positive	28 (65.1)	14	14		17	11	

**Table 2:** Univariate and multivariate analyses of RFS in SCCE patients.

	Survival (%)	Chi-square	*P* value	HR (95% CI)	*P* value
Age (years)		0.883	0.347		
≤60	20.8				
>60	21.1				
Gender		0.937	0.333		
Female	23.1				
Male	20.0				
Tumor length (cm)		3.901	0.048		0.680
≤3	27.8			1.000	
>3	16.0			1.187 (0.525–2.688)	
Tumor location		1.775	0.183		
Upper/middle	32.0				
Lower	5.6				
Vessel involvement		2.122	0.145		
Negative	24.2				
Positive	10.0				
Depth of invasion		8.439	0.004		0.255
T1-2	38.9			1.000	
T3-4a	8.0			1.656 (0.695–3.941)	
Nodal metastasis		11.376	0.001		0.019
Negative	40.0			1.000	
Positive	10.7			3.219 (1.216–8.520)	
Chemoradiotherapy		2.834	0.092		
No	17.6				
Yes	23.1				
NLR		1.879	0.170		
<3.5	22.2				
≥3.5	18.8				
PLR		5.027	0.025		0.083
<150	25.0			1.000	
≥150	13.3			1.999 (0.915–4.367)	

**Table 3:** Univariate and multivariate analyses of OS in SCCE patients.

	Survival (%)	Chi-square	*P* value	HR (95% CI)	*P* value
Age (years)		0.686	0.408		
≤60	16.7				
>60	21.1				
Gender		1.841	0.175		
Female	38.5				
Male	10.0				
Tumor length (cm)		6.846	0.009		0.627
≤3	33.3			1.000	
>3	8.0			1.229 (0.535–2.821)	
Tumor location		0.024	0.877		
Upper/middle	20.0				
Lower	16.7				
Vessel involvement		1.068	0.301		
Negative	21.2				
Positive	10.0				
Depth of invasion		7.433	0.006		0.103
T1-2	33.3			1.000	
T3-4a	8.0			2.032 (0.867–4.766)	
Nodal metastasis		9.687	0.002		0.092
Negative	40.0			1.000	
Positive	7.1			2.182 (0.880–5.411)	
Chemoradiotherapy		5.577	0.018		0.011
No	5.9			1.000	
Yes	26.9			0.380 (0.180–0.803)	
NLR		1.967	0.161		
<3.5	22.2				
≥3.5	12.5				
PLR		7.374	0.007		0.041
<150	25.0			1.000	
≥150	6.7			2.272 (1.035–4.984)	
